# Factors Related to Evidence-Based Practices Among Mental Health Nurses in Thailand: A Cross-Sectional Study

**DOI:** 10.3390/nursrep14040224

**Published:** 2024-10-18

**Authors:** Napapat Manitkul, Kwaunpanomporn Thummathai, Patraporn Bhatarasakoon

**Affiliations:** 1 Faculty of Nursing, Chiang Mai University, 110/406, Suthep, Muang Chiang Mai, Chiang Mai 50200, Thailand; napapat_m@cmu.ac.th (N.M.); kwaunpanomporn.th@cmu.ac.th (K.T.); 2 The Thailand Centre for Evidence-Based Health Care, Faculty of Nursing, Chiang Mai University, Chiang Mai 50200, Thailand

**Keywords:** evidence-based practice, attitude, knowledge/skills, organizational culture, mentorship, demographic factors, mental health nurses, Thailand, nursing

## Abstract

Background and Objectives: Despite the robustness of evidence-based practice in increasing efficiency in nursing care and improving patient safety, using evidence in practice is still rare in mental health nursing. This correlational descriptive research aimed to explore the factors and examine the relationship between attitudes, knowledge/skills, organizational culture, mentorship, and demographic factors to evidence-based practices among mental health nurses working in psychiatric hospitals in Thailand. Methods: The sample consisted of 255 nurses working in psychiatric hospitals under the Thai Department of Mental Health, located in service units across the country’s four regions. The data collection tools included (1) a demographic questionnaire, (2) the Evidence-Based Practice Questionnaire: EBPQ, (3) Organizational Culture & Readiness for System-wide Integration of Evidence-Based Practice: OCRSIEP, and (4) the Evidence-Based Practice Mentorship Scale. Descriptive statistics and Spearman’s correlation coefficient were used for data analysis. Results: The findings revealed that the factors positively correlated with evidence-based practice among mental health nurses in Thailand were attitude with a mean score of 4.90 (r = 0.39, *p*-value < 0.001), knowledge/skills with a mean score of 4.69 (r = 0.61, *p*-value < 0.001), organizational culture with a mean score of 3.94 (r = 0.26, *p*-value < 0.001), and mentorship with a mean score of 2.77 (r = 0.16, *p*-value = 0.011). Demographic factors such as educational level (r = 0.21, *p*-value < 0.001) and work experience in psychiatric and mental health nursing (r = 0.14, *p*-value = 0.031) were also positively correlated. Conclusions: This research revealed that EBP knowledge and skills are the most significant factors related to evidence-based practice among Thai mental health nurses. Therefore, EBP knowledge and skills should be enhanced in the curriculum during the nursing study and through continuing education once nurses graduate. Organizational culture and mentorship also need to be promoted to strengthen the use of EBP in Thailand.

## 1. Introduction

Evidence-based practice is a framework for increasing efficiency in nursing. It interplays the use of current best evidence with reliable research studies combined with clinical expertise, values, and patient needs [1]. Evidence-based practices increase patient safety, improve treatment outcomes, and reduce healthcare costs [2]. Although evidence-based practice is important, it was found that there were still obstacles to using research evidence in nursing practice, including the implementation and level of knowledge of nurses [3]. The World Health Organization states that nursing practice should be based on the best available evidence [4]. However, implementing evidence-based practice in mental health nursing to care for patients is challenging. Mental health nurses rarely follow evidence-based practices in their work. Although evidence-based practice is valued and accepted, mental health nurses need help integrating it into their nursing activities. They tend to rely more on traditional nursing practices than evidence-based practice [5]. A study in Saudi Arabia [6] shows that mental health nurses believe in the importance of evidence-based practice and its positive impact on patient outcomes; however, they face various barriers, such as insufficient time for research, lack of support and guidelines within organizations, and difficulties in understanding English-language research. Therefore, mental health nurses rely on their accumulated experiences to care for patients.

Many factors affect nurses’ use of evidence in nursing practice, such as attitudes, knowledge/skills, organizational culture, mentorship, competency, and beliefs [2]. The literature revealed that factors related to practice based on empirical evidence, which are used in this study, include attitudes, knowledge/skills, organizational culture, mentorship, and demographic characteristics, including age, education level, and work experience [2,7,8]. Most of the factors mentioned are positively related to evidence-based practice, including attitude [7,9], knowledge/skills [10,11,12], organizational culture [13,14,15,16], and mentorship [17,18]. Age [2,7], education level [2,19,20], and work experience [20] have an unclear relationship with evidence-based practice, indicating the need to study these factors to reach a conclusion.

Recent studies have been conducted on evidence-based practices among advanced nurse practitioners in Thailand [7]. However, these studies only provide an overview of the results of evidence-based practice in advanced nurse practitioners and do not focus on specific evidence-based practices in mental health nursing. Specialized nursing care for mental health patients requires evidence-based practice that is current and up to date. The context and organizational culture of nursing for mental health patients differs from that of general patients, so factors involved in evidence-based practice among mental health nurses may vary accordingly.

This study aimed to explore the factors and examine the relationship between factors that affect evidence-based practice among mental health nurses in Thailand. The information obtained will be used for planning, developing, and promoting evidence-based nursing practice for mental health nurses in Thailand.

## 2. Materials and Methods

This study uses a descriptive correlational research approach to explore and examine relationships among factors associated with evidence-based practice in mental health nursing in Thailand.

### 2.1. Setting

From December 2023 to January 2024, a survey was conducted among mental health nurses in 20 service units in psychiatric hospitals under the Department of Mental Health in Thailand. The service includes mental health treatment and care for inpatients, outpatients, patients with complicated mental illnesses, and rehabilitation for chronic mental health patients.

### 2.2. Sampling

The sample for the study was randomly selected to accurately reflect the population proportions of all four regions. There were 20 service units across four regions, and a single service unit was randomly chosen from each region using simple random sampling. The selected service units included the Rajanagarindra Institute of Child Development in the north, the Nakhon Ratchasima Rajanagarindra Psychiatric Hospital in the northeast, the Yuwaprasart Waithayopathum Child and Adolescent Psychiatric Hospital in the central, and the Suansaranrom Hospital in the south region. The four randomly selected hospitals, one for each region, collectively employed 432 nurses, with 38, 120, 44, and 230, respectively. The Finite Population Proportion formula was utilized to calculate the sample size proportion [21]. The sample size calculated was 204 nurses. To prevent loss or incomplete responses, the sample size was increased by 20% of the sample [22]. Therefore, a total of 255 nurses were included in this study. Using the proportional method, the sample required from each hospital were 22, 71, 26, and 136, respectively. The inclusion criteria for the sample required the nurses to agree to participate in the research project and to have had at least one year of experience providing psychiatric services in a hospital or psychiatry institute eligible to be included in this study.

### 2.3. Instruments

The data was obtained through a questionnaire that was divided into four sections.

#### 2.3.1. Demographic Questionnaire

The demographic questionnaire includes gender (male and female), age, education level, and work experience.

#### 2.3.2. Evidence-Based Practice Questionnaire (EBPQ)

EBPQ, developed by Upton and Upton in 2006 [23], measured attitude, knowledge/skills, and practice towards evidence-based practice with a 7-Point-Likert-type scale. It was divided into four attitude questions, fourteen knowledge/skills questions, and six practice questions, with a total of twenty-four questions. Higher scores reflect higher levels of practice, attitude, and knowledge/skills for evidence-based practice. The Thai version of EBPQ used in this study has been translated by Patraporn Bhatarasakoon and Somchit Hanucharurnkul, validated and used in a Thai advanced practice nurse study [7]. The reliability was tested with an additional 30 mental health nurses similar to the sample and yielded a Cronbach’s alpha coefficient of 0.97

#### 2.3.3. Organizational Culture & Readiness for System-Wide Integration of Evidence-Based Practice (OCRSIEP)

OCRSIEP was developed and yielded a good psychometric property by Melnyk, Hsieh, and Mu [24]. This instrument taps the extent to which cultural factors influence the system-wide implementation of EBP in the organization and the overall perceived readiness for integrating EBP. Responses were designed using a 5-Point-Likert-type scale, ranging from 1 = not at all to 5 = very much. Total scores range from 25 to 125. Higher scores indicate the readiness of the organizational culture for evidence-based practice. The OCRSIEP was translated from the original English version into Thai using a back-translation procedure and then reviewed by experts to ensure consistency of meaning between the two languages. The Content Validity Index (CVI) was found to be 0.96, deemed acceptable. The Thai version of OCRSIEP was then piloted with an additional 30 mental health nurses similar to the sample. The overall Cronbach’s alpha coefficient was 0.94.

#### 2.3.4. EBP Mentorship Scale

The EBP Mentorship Scale was developed by Melnyk, Hsieh, and Mu [25]. This instrument taps the degree of EBP mentorship available to nurses. Responses were designed using an 8-item Likert-type scale, ranging from 0 = none at all to 4 = very much so. Total scores range from 0 to 32. Higher scores indicate higher mentorship. The EBP Mentorship Scale was translated from the original English into Thai using a back-translation procedure and then reviewed by experts to ensure consistency of meaning between the two languages. The Content Validity Index (CVI) was found to be 1, deemed acceptable. The Thai version of the EBP Mentorship Scale was then piloted with an additional 30 mental health nurses similar to the sample. The overall Cronbach’s alpha coefficient was 0.98.

### 2.4. Data Collection

The researcher approached eligible participants to introduce the project and its objectives and answer any questions. A QR code was provided to them when they expressed their interest. Participants were required to provide their consent to participate in the research online. They were given a QR code to scan to enter the consent form page first. If they agreed to participate and provided the consent form, then they would go to the page of the questionnaire and complete the questionnaire in a Google Form. The questionnaires were to be collected within one week from the online platform. A total of 255 nurses completed the questionnaires and submitted them online (response rate = 100%).

### 2.5. Ethical Considerations

Ethical approval was obtained from the authors’ institute with study code 2566-EXP028 (29 May 2023) and data collection settings IRB with study code DMH.IRB 021/2566 BRm_Ful (4 September 2023). All mental health nurses participating in this study provided their consent forms. All personal information was kept confidential.

### 2.6. Statistical Analysis

The Statistical Package for Social Sciences (SPSS) version 29 was used to analyze the data. Descriptive statistics were employed to measure frequencies, percentages, means, and standard deviations. Relationships between variables were examined using Spearman’s rho. The significance level was at 0.05.

## 3. Results

### 3.1. Description of Demographic Data

The final sample comprised 255 mental health nurses; 224 (87.80%) were females, and 31 (12.20%) were males. The average age was 43.19, SD = 11.30. One out of a third of the sample graduated from specialized nursing courses in mental health and psychiatric nursing (38%). More than half of the sample had work experience as a nurse of more than or equal to 16 years (54.50%), and 46.70% of the sample had work experience in psychiatric and mental health nursing of more than or equal to 16 years. Details are shown in Table 1.

### 3.2. Description of Attitude

Attitude was found to have an overall mean of 4.90 out of 7 points, which was separated into individual questions. The question with the highest average was “I stick to tried and trusted methods rather than changing to anything new vs my practice has changed because of evidence I have found” followed by “My workload is too great for me to keep up to date with all the new evidence vs new evidence is so important that I make the time in my work schedule”, and the question with the lowest average was “Evidence-based practice is a waste of time vs evidence-based practice is fundamental to professional practice”. Details are shown in Table 2.

### 3.3. Description of Knowledge/Skills

Knowledge/skills was found to have an overall mean of 4.69 out of 7 points, which was separated into individual questions. The question with the highest average was “Ability to review your own practice” followed by “Sharing of ideas and information with colleagues”, and the question with the lowest average was “Research skills”. Details are shown in Table 3.

### 3.4. Description of Organizational Culture

Organizational culture was found to have an overall mean of 3.94 out of 5 points, which was separated into individual questions. The question with the highest average was “To what extent is EBP clearly described as central to the mission and philosophy of your institution?” followed by “To what extent is the physician team with whom you work committed to EBP?” and the question with the lowest average was “To what extent are librarians used to search for evidence?” Details are shown in Table 4.

### 3.5. Description of Mentorship

Mentorship was found to have an overall mean of 2.77 out of 4 points, which was separated into individual questions. The question with the highest average was “I have access to a mentor who assists me with implementing the seven steps of evidence-based practice” followed by “I have a mentor who is consistently available to me when I have questions about evidence-based practice” and “Mentorship is available here to assist me with making practice changes based on best evidence”, and the question with the lowest average was “Mentorship is available here to assist me in rapid critical appraisal of studies and synthesis of evidence”. Details are shown in Table 5.

### 3.6. Description of Practice

Practice was found to have an overall mean of 5.16 out of 7 points, measured by six items from the EBPQ practice component. The question with the highest average was “Shared this information with colleagues” followed by “Formulated a clearly answerable question as the beginning of the process towards filling this gap”, and the question with the lowest average was “Critically appraised, against set criteria, any literature you have discovered”. Details are shown in Table 6.

### 3.7. Relationships of Demographic Factors, Attitude, Knowledge/Skills, Organizational Culture, and Mentorship with Evidence-Based Practice

Relationships between factors related to evidence-based practice among mental health nurses in Thailand include demographic characteristics, attitudes, knowledge/skills, organizational culture, and mentorship. The results showed knowledge/skills had a high positive correlation with practice (r = 0.61, *p* < 0.001); age and working experience as a nurse were not correlated to EBP. Details are shown in Table 7.

## 4. Discussion

The study found that the average attitude level was 4.90, with a positive relationship between attitude and evidence-based practice among mental health nurses (r_s_ = 0.39, *p* < 0.00). It was found that mental health nurses in Thailand agreed with “my practice has changed because of evidence I have found” with an average score as high as 5.02. This was consistent with the research previously conducted in Thailand [9], China [26], and Iran [27]. The findings indicate that when mental health nurses maintain a positive attitude toward evidence-based practice, it significantly changes the implementation of evidence-based practices.

Knowledge and skills are highly positively related to evidence-based practice among mental health nurses in this study (r_s_ = 0.61, *p* < 0.00), with a mean score of 4.69. If mental health nurses possess knowledge and skills in evidence-based practice, this can lead to an increase in evidence-based practice. According to studies conducted in Iran [28], Malawi [29], and Malaysia [30], this finding is consistent. However, considering each item, it was found that the mental health nurses in Thailand scored the lowest in “Research skills” and “Converting your information needs into a research question” with an average score of 4.01 and 4.18, respectively. This finding is consistent with previous research conducted in Thailand [7] and the USA [31]. Therefore, mental health nurses in Thailand should be prepared more in terms of their research knowledge, especially the skills to transform data into research questions. This is considered the first step and an important factor when they need to sort out the evidence to answer their questions when faced with clinical problems. Also, knowledge and skills were found to have the highest correlation among other factors in this study, which highlights their importance in implementing EBP.

The study reveals the positive correlation between mental health nurses’ evidence-based practice and organizational culture (r_s_ = 0.26, *p* < 0.00) with a mean score of 3.94. If mental health nurses work in a unit where the organizational culture is prepared for evidence-based practice, they are more likely to succeed. The key to success is having an environment ready for this approach. Studies conducted in South Korea [13] found a positive relationship between organizational readiness and evidence-based nursing practice. The study results suggest that an organization’s readiness level is the most important factor in determining the adoption of evidence-based practice. A culture for evidence-based practice in nursing organizations can be established by creating policies, a vision, a budget, and support for implementation. A study in the USA [32] also found that organizational culture and readiness were positively related to evidence-based practice. If the organization is prepared to change and create an environment conducive to evidence-based practice, nurses’ confidence in practicing evidence-based care will increase. A study by Galuska et al. [33] found that perceptions of organizational culture and readiness for evidence-based practice were positively related to evidence-based practice. There must be a vision and a model for evidence-based practice for it to become a culture within the organization. Providing the necessary support and investment is important in implementing evidence-based practices effectively. Based on the study’s findings, a positive organizational culture can facilitate the implementation of evidence-based practice among mental health nurses. This is because an agency with clear policies, a strong vision, and robust support from within the organization is more likely to have the necessary resources, such as budgets, equipment, and human resources, to promote evidence-based practice. Therefore, organizational culture is considered a crucial factor in strengthening evidence-based practice within an organization, ensuring its sustainability.

It was found that mentorship positively correlated with evidence-based practice among mental health nurses (r_s_ = 0.16, *p* < 0.01), with an average of 2.77. If mental health nurses receive evidence-based practice mentorship, they will increase their use of evidence-based practice. Similar to research in the USA [15,34], our findings indicate that mentorship has a positive relationship with evidence-based practice. If mentorship is available, it can help increase the implementation of evidence-based practices. According to the study, mentorship is an effective way to promote evidence-based practice. If someone can guide and recommend evidence-based practice, mental health nurses will feel more confident. Unfortunately, mentorship for evidence-based practices in Thailand remains rare in many departments. It is necessary to provide support and promotion to experts or agencies who can offer advice to mental health nurses within the organization.

Regarding the demographic variables, a study found that age was unrelated to evidence-based practice among mental health nurses. This was consistent with the studies conducted in Thailand [7], Jordan [35], and Norway [36]. However, in Saudi Arabia [37], studies have shown that younger nurses practice evidence-based practice more often than older nurses. This included a Bell (2020) [38] study that found that younger nurses were more strongly related to their ability to practice evidence-based practice. Therefore, younger nurses practice more evidence-based practices than older nurses. It is likely that younger nurses are more open to new things and more prepared to practice evidence-based practices than older nurses. It is unclear whether age alone can explain the link with evidence-based adherence. However, promoting and implementing strategies that support evidence-based practice among mental health nurses of all ages can improve the overall level of evidence-based practice.

The next factor is education level. The study found that education level was positively related to evidence-based practice among mental health nurses (r_s_ = 0.21, *p* < 0.00). Consistent with a study in Israel [39], education level was found to be associated with evidence-based practice. Nurses with a higher degree are more likely to practice evidence-based practice than nurses with a lower degree. Studies in Jordan [40] and Iran [41] have found that nurses with higher education tend to practice more evidence-based practices than nurses with lower education levels. Therefore, education level has an impact on evidence-based practice. Suppose mental health nurses increase their knowledge and develop themselves by continuing their education at a higher level. In that case, it will result in more evidence-based practice based on the knowledge gained from their studies.

The study results found that work experience as a nurse was not related to evidence-based practice. On the contrary, work experiences in psychiatric and mental health nursing found a positive relationship with evidence-based practice among mental health nurses (r_s_ = 0.14, *p* < 0.031). Previous studies have indicated that work experiences vary significantly. A study conducted in Oman [42] revealed a positive correlation between nurses’ experience and evidence-based practice. According to research conducted in Thailand, nurses with greater work experience are more likely to engage in evidence-based practice [7]. Opposite results were found in Saudi Arabia [43] and Jordan [35] regarding the relationship between work experience and evidence-based practice among nurses. In Saudi Arabia, a study of 227 nurses found no correlation between work experience and evidence-based practice. Similarly, in Jordan, a study of 500 nurses found that work experience was not related to evidence-based practice. Therefore, experience as a nurse or in psychiatric and mental health nursing does not definitively explain the relationship with evidence-based practice. Encouraging continuous and consistent evidence-based practice is important for individual mental health nurses with varying levels of work experience to provide efficient patient care in the future.

## 5. Conclusions

This study indicates that knowledge/skills, attitude, and organizational culture are key factors that enhance the evidence-based practice of mental health nurses in Thailand.

Mental health nurses must receive more promotion of research skills to support evidence-based practice without delay. Research skills are a crucial part of providing effective care to patients with mental health issues. Therefore, it is imperative to equip mental health nurses with the necessary knowledge and skills to conduct research and apply its results in their practice. Cultivating a positive attitude towards evidence-based practice among mental health nurses can lead to changes in their nursing practice. Knowledge regarding evidence-based practices is shared both within and across agencies. This leads to the planning and establishing policies to ensure that evidence-based practices are followed at an organizational level. Ultimately, this makes treatment safer and more satisfying for patients.

## Figures and Tables

**Table 1:** Demographic data (N = 255).

Variable	Number (Person)	Percentage
Gender		
Male	31	12.20
Female	224	87.80
Age (years)		
20–29 years	27	10.60
30–39 years	90	35.30
40–49 years	53	20.80
Age greater than or equal to 50 years (range = 23–60 years, mean = 43.19, SD = 11.30)	85	33.30
Education level		
Graduated with a Bachelor of Nursing Science degree	73	28.60
Completed a specialized nursing course in mental health and psychiatric nursing	97	38.00
Graduated with a Master of Nursing Science degree in Psychiatric and Mental Health Nursing	72	28.20
Graduated with a Master of Nursing Science or a master’s degree in a related field	9	3.50
Completed the Advanced Practice Nurse (APN) program in Psychiatric and Mental Health Nursing (42 credits)	2	0.80
Completed the Advanced Practice Nurse (APN) training course in Psychiatric and Mental Health Nursing (3-years program equivalent to a doctorate degree)	2	0.80
Work experience as a nurse		
Less than 5 years	21	8.20
6–10 years	53	20.80
11–15 years	42	16.50
More than or equal to 16 years (range = 2–43 years, mean = 20.35, SD = 11.85)	139	54.50
Work experience in psychiatric and mental health nursing		
Less than 5 years	52	20.40
6–10 years	21	20.00
11–15 years	33	12.90
More than or equal to 16 years (range = 1–40 years, mean = 16.99, SD = 11.76)	119	46.70

**Table 2:** Mean scores and standard deviation for attitude (N = 255).

Attitude	Mean	SD
Overall attitude	4.90	1.38
Attitude subscale		
1. My workload is too great for me to keep up to date with all the new evidence vs new evidence is so important that I make the time in my work schedule	5.01	1.34
2. I resent having my clinical practice questioned vs I welcome questions on my practice	4.83	1.72
3. Evidence-based practice is a waste of time vs evidence-based practice is fundamental to professional practice	4.73	2.00
4. I stick to tried and trusted methods rather than changing to anything new vs my practice has changed because of evidence I have found	5.02	1.52

**Table 3:** Mean scores and standard deviation for knowledge/skills (N = 255).

Knowledge/Skills	Mean	SD
Overall knowledge/skills	4.69	1.19
Knowledge/skills subscale		
1. Research skills	4.01	1.47
2. Information technology skills	4.60	1.25
3. Monitoring and reviewing of practice skills	4.93	1.32
4. Converting your information needs into a research question	4.18	1.48
5. Awareness of major information types and sources	4.83	1.40
6. Ability to identify gaps in your professional practice	4.69	1.30
7. Knowledge of how to retrieve evidence	4.69	1.34
8. Ability to analyze critically evidence against set standards	4.56	1.38
9. Ability to determine how valid (close to the truth) the material is	4.53	1.39
10. Ability to determine how useful (clinically applicable) the material is	4.69	1.32
11. Ability to apply information to individual cases	4.90	1.33
12. Sharing of ideas and information with colleagues	5.03	1.32
13. Dissemination of new ideas about care to colleagues	4.92	1.34
14. Ability to review your own practice	5.07	1.33

**Table 4:** Mean scores and standard deviation for organizational culture (N = 255).

Organizational Culture	Mean	SD
Overall organizational culture	3.94	0.57
Organizational culture subscale		
1. To what extent is EBP clearly described as central to the mission and philosophy of your institution?	4.48	0.65
2. To what extent do you believe that EBP is practiced in your organization?	4.32	0.64
3. To what extent is the nursing staff with whom you work committed to EBP?	4.25	0.70
4. To what extent is the physician team with whom you work committed to EBP?	4.38	0.65
5. To what extent are there administrators within your organization committed to EBP (i.e., have planned for resources and support [e.g., time] to initiate EBP)?	4.28	0.70
6. In your organization, to what extent is there a critical mass of nurses who have strong EBP knowledge and skills?	4.12	0.76
7. To what extent are there nurse scientists (doctorally prepared researchers) in your organization to assist in generation of evidence when it does not exist?	3.45	1.06
8. In your organization, to what extent are there Advanced Practiced Nurses who are EBP mentors for staff nurses as well as other APNs?	3.58	0.99
9. To what extent do practitioners model EBP in their clinical settings?	4.08	0.70
10. To what extent do staff nurses have access to quality computers and access to electronic databases for searching for best evidence?	4.07	0.84
11. To what extent do staff nurses have proficient computer skills?	4.21	0.65
12. To what extent do librarians within your organization have EBP knowledge and skills?	3.30	1.27
13. To what extent are librarians used to search for evidence?	3.18	1.25
14. To what extent are fiscal resources used to support EBP (e.g., education, attending EBP conferences/workshops, computers, paid time for the EBP process, mentors)?	3.76	0.89
15. To what extent are there EBP champions (i.e., those who will go the extra mile to advance EBP) in the environment among:		
15.1. Administrators?	3.73	1.04
15.2. Physicians?	3.81	1.00
15.3. Nurse Educators?	3.81	1.09
15.4. Advance Nurse Practitioners?	3.83	0.98
15.5. Staff Nurses?	3.74	0.96
16. To what extent is the measurement and sharing of outcomes part of the culture of the organization in which you work?	4.13	0.78
17. To what extent are decisions generated from:		
17.1. Direct care providers?	3.96	0.85
17.2. Upper administration?	3.89	0.77
17.3. Physician or other healthcare provider groups?	4.04	0.77
18. Overall, how would you rate your institution in readiness for EBP?	4.05	0.76
19. Compared to 6 months ago, how much movement in your organization has there been toward an EBP culture?	4.11	0.78

**Table 5:** Mean scores and standard deviation for mentorship (N = 255).

Mentorship	Mean	SD
Overall mentorship	2.77	0.80
Mentorship subscale		
1. I have access to a mentor who assists me with implementing the seven steps of evidence-based practice	2.84	0.88
2. Mentorship is available here to assist me in forming PICOT questions	2.74	0.90
3. Mentorship is available here to assist me in rapid critical appraisal of studies and synthesis of evidence	2.70	0.83
4. I have a mentor who is consistently available to me when I have questions about evidence-based practice	2.80	0.88
5. Mentorship is available here to assist me with making practice changes based on best evidence	2.80	0.89
6. I have access to a mentor here to assist me with evaluating practice outcomes	2.79	0.87
7. Mentorship is available here to assist me in disseminating evidence through presentations and publications	2.72	0.89
8. I have access to a mentor here who encourages me to question my practice and engage in EBP	2.78	0.85

**Table 6:** Mean scores and standard deviation for practice (N = 255).

Practice	Mean	SD
Overall practice	5.16	1.06
Practice subscale		
1. Formulated a clearly answerable question as the beginning of the process towards filling this gap	5.29	1.13
2. Tracked down the relevant evidence once you have formulated the question	5.06	1.24
3. Critically appraised, against set criteria, any literature you have discovered	4.84	1.30
4. Integrated the evidence you have found with your expertise	5.07	1.27
5. Evaluated the outcomes of your practice	5.26	1.24
6. Shared this information with colleagues	5.42	1.20

**Table 7:** Associations of demographic factors, attitude, knowledge/skills, organizational culture, and mentorship with practice (N = 255).

Variable	Correlation Coefficient	*p*-Value	Result
Demographic factors			
Age	r_s_ = 0.07	0.266	Not correlated
Education level	r_s_ = 0.21	<0.001	Low positive correlation
Work experience as a nurse	r_s_ = 0.07	0.296	Not correlated
Work experience in psychiatric and mental health nursing	r_s_ = 0.14	0.031	Very low positive correlation
Attitude	r_s_ = 0.39	<0.001	Low positive correlation
Knowledge/skills	r_s_ = 0.61	<0.001	High positive correlation
Organizational culture	r_s_ = 0.26	<0.001	Low positive correlation
Mentorship	r_s_ = 0.16	0.011	Very low positive correlation

r_s_ is Spearman’s rho.

## Data Availability

Data are available from the corresponding author upon request.

## Public Involvement Statement

Mental health nurses were involved. They completed a self-administered questionnaire.

## Guidelines and Standards Statement

This manuscript adheres to STROBE guidelines for observational studies.
